# Trace Metal Contamination Impacts Predicted Functions More Than Structure of Marine Prokaryotic Biofilm Communities in an Anthropized Coastal Area

**DOI:** 10.3389/fmicb.2021.589948

**Published:** 2021-02-19

**Authors:** Clément Coclet, Cédric Garnier, Sébastien D’Onofrio, Gaël Durrieu, Emilie Pasero, Christophe Le Poupon, Dario Omanović, Jean-Ulrich Mullot, Benjamin Misson, Jean-François Briand

**Affiliations:** ^1^Université de Toulon, Laboratoire MAPIEM, EA 4323, Toulon, France; ^2^Université de Toulon, Aix Marseille Université, CNRS, IRD, Mediterranean Institute of Oceanography, UM110, La Garde, France; ^3^Microbia Environnement Observatoire Océanologique, Banyuls-sur-Mer, France; ^4^Division for Marine and Environmental Research, Ruðer Bošković Institute, Zagreb, Croatia; ^5^LASEM-Toulon, Base Navale De Toulon, BP 61, Toulon, France

**Keywords:** prokaryotic biofilms, bacterioplankton, trace metal contamination, Illumina Miseq sequencing, marine coastal environment

## Abstract

Trace metal (TM) contamination in marine coastal areas is a worldwide threat for aquatic communities. However, little is known about the influence of a multi-chemical contamination on both marine biofilm communities’ structure and functioning. To determine how TM contamination potentially impacted microbial biofilms’ structure and their functions, polycarbonate (PC) plates were immerged in both surface and bottom of the seawater column, at five sites, along strong TM contamination gradients, in Toulon Bay. The PC plates were incubated during 4 weeks to enable colonization by biofilm-forming microorganisms on artificial surfaces. Biofilms from the PC plates, as well as surrounding seawaters, were collected and analyzed by 16S rRNA amplicon gene sequencing to describe prokaryotic community diversity, structure and functions, and to determine the relationships between bacterioplankton and biofilm communities. Our results showed that prokaryotic biofilm structure was not significantly affected by the measured environmental variables, while the functional profiles of biofilms were significantly impacted by Cu, Mn, Zn, and salinity. Biofilms from the contaminated sites were dominated by tolerant taxa to contaminants and specialized hydrocarbon-degrading microorganisms. Functions related to major xenobiotics biodegradation and metabolism, such as methane metabolism, degradation of aromatic compounds, and benzoate degradation, as well as functions involved in quorum sensing signaling, extracellular polymeric substances (EPS) matrix, and biofilm formation were significantly over-represented in the contaminated site relative to the uncontaminated one. Taken together, our results suggest that biofilms may be able to survive to strong multi-chemical contamination because of the presence of tolerant taxa in biofilms, as well as the functional responses of biofilm communities. Moreover, biofilm communities exhibited significant variations of structure and functional profiles along the seawater column, potentially explained by the contribution of taxa from surrounding sediments. Finally, we found that both structure and functions were significantly distinct between the biofilm and bacterioplankton, highlighting major differences between the both lifestyles, and the divergence of their responses facing to a multi-chemical contamination.

## Introduction

Trace metal (TM) pollution is a global concern in marine environments due to and currently increasing metal emissions (The Mermex Group et al., 2011) but also the legacy of historic contamination. Coastal areas of the Mediterranean Sea are traditionally zones of intense human activities, characterized by highly urbanized and industrialized coastline with large harbors and major cities. In this context, pollution is increasingly present as a result of anthropogenic activities, making marine coasts, one of the most heavily contaminated aquatic environments by TMs (Förstner and Wittmann, 2012; de Souza Machado et al., 2016). Thus, TM concentrations in Mediterranean coastal surface waters, mainly due to atmospheric inputs, are higher than those, for example, in Atlantic Ocean (Heimbürger et al., 2011; Cossa et al., 2020). Rather than the atmospheric pathway, TM seawater contamination of the semi-enclosed Toulon Bay (Mediterranean Sea, France) is primarily caused by urban and industrial wastes (Levin et al., 2001; Oursel et al., 2013), antifouling coatings (Turner, 2010), fuel consumption (Callender, 2003), and sediment resuspension (Dang et al., 2015; Layglon et al., 2020). Then, seawater of the Toulon Bay is characterized by high levels of anthropogenic (Cd, Pb, and Zn) and intermediate (i.e., both anthropogenic and natural origins) (Cu and Ni) TMs (Jean et al., 2012; Coclet et al., 2018, 2019, 2020; Layglon et al., 2020), which can be 100-fold above geochemical background levels of the Mediterranean Sea (Morley et al., 1997), and by far, upper than most of Mediterranean coastal zones (Cossa et al., 2020).

Biofilms are sessile communities formed by microbes, which are able to colonize any submerged surface and form complex structure over time, as defined by the cell attachment and production of a hydrated polymeric matrix that allows aggregation (Costerton, 1995; Dang and Lovell, 2016). Because microbial biofilm communities were shown to be influenced by some local environmental conditions (Lee et al., 2014; Briand et al., 2017; Catão et al., 2019; Caruso, 2020), biofilms could also be significantly affected by TM contamination encountered in coastal areas (Corcoll et al., 2019). In this sense, the structure of microbial biofilms from contaminated areas have been shown to present divergence with microbial biofilms from uncontaminated areas (Webster and Negri, 2006; Briand et al., 2012; McElroy et al., 2016; Pollet et al., 2018). Several studies showed that exposure to antifouling coating containing TMs reduced biofilm biomass as well as altered both formation and structure of microbial biofilm (Camps et al., 2014; Catão et al., 2019; Liang et al., 2019). A number of studies have indicated that TMs have toxicological effects against exposed microbes into marine biofilms (Corcoll et al., 2019), through the alteration of genes important to cellular processes or metabolic activities related to photosynthesis, nitrogen cycling, degradation and biosynthesis of lipids (Ding et al., 2019; Bergsveinson et al., 2020). However, relationships between both structure and function of microbial biofilm communities, and TM contamination in coastal and especially harbor environment remains to be determined.

Nevertheless, biofilm lifestyle provide to microbes, numerous ecological advantages, including greater access to nutritional resources, enhanced organism interactions, and environmental stability (Costerton, 1995; Decho, 2000; McDougald et al., 2011; Salta et al., 2013; Dang and Lovell, 2016). It is frequently observed that bacterial biofilms can withstand the effects of toxic metals better than planktonic cultures of the same species (Teitzel and Parsek, 2003; Harrison et al., 2007). It has been also reported that the increased production of extracellular polymeric substances (EPS) helps to increase the resistance of biofilms to heavy metals because EPS can absorb a lot of heavy metals, thereby reducing its toxicity to microbial cells (Lin et al., 2020). The presence of a small population of persistent cells into the biofilm structure may contribute to the tolerance of microbial biofilms to TM contamination (Harrison et al., 2005). These findings suggested existence of ubiquitous mechanisms of resistance and tolerance to TM, governing biofilm formation on surface in contaminated environments (Dang and Lovell, 2002, 2016; Ding et al., 2019). Thereby, biofilm constitutes a major microbial lifestyle highlighting the ecological success of biofilms as habitat formers (Flemming et al., 2016). Although a quantity of studies has reported the interactions between metal surface types and both the biomass and the structure of microbial biofilms (Richard et al., 2019; Caruso, 2020), the diversity of microbial biofilms subject to TM contamination in the environment remains largely elusive. As biofilms contribute to deleterious effects by reducing hydrodynamic performance and increasing fuel consumption when colonizing ship hulls (Schultz et al., 2011) or promoting the corrosion of metallic structures like off-shore platforms or marine renewable facilities (Païssé et al., 2013; Kip and van Veen, 2015; Macleod et al., 2016), they become a prominent marine environmental issue (Zettler et al., 2013; Debroas et al., 2017; Dussud et al., 2018; Oberbeckmann et al., 2018). Moreover, as sessile integrative systems, biofilms were also promoted as bioindication tools to monitor pollution, almost exclusively in freshwater environment (Tlili et al., 2016; Weigand et al., 2019).

In the present study, using both flow cytometry and 16S rRNA gene amplicon sequencing, we examined prokaryotic biofilms formed on polycarbonate (PC) plates and surrounding bacterioplankton community in five sites at the surface and bottom of the seawater column, along the chemical contamination gradient in Toulon Bay. We hypothesized that clear difference could be observed between prokaryotic biofilm and bacterioplankton communities, and microbes in biofilms may adopt certain mechanisms of resistance or tolerance to TM contamination. To test these hypothesizes, we (i) characterized taxonomic and functional patterns of prokaryotic biofilm communities across TM contamination gradients at five sites in the Toulon Bay, (ii) assess the potential effect of depth on biofilm community at the same sites, and (iii) investigated relationships between bacterioplankton and biofilm communities in this context.

## Materials and Methods

### Study Area, Experimental Design, and Sample Collection

**FIGURE 1:**
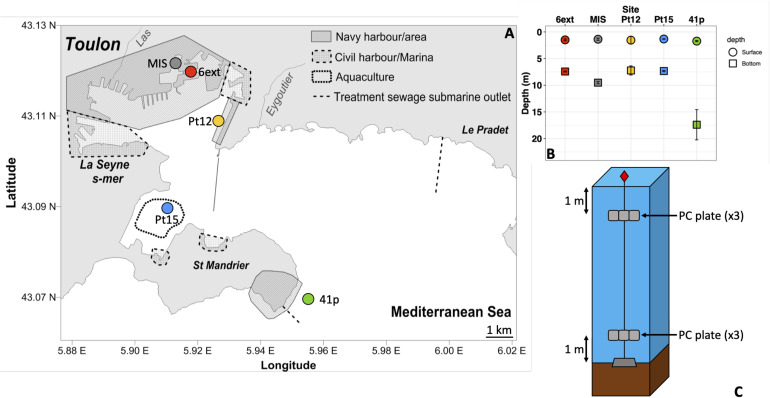
Map of the Toulon Bay with the location of the sampling sites **(A)**. Solid and dashed lines represent a seawall and treated sewage submarine outlets, respectively. Bottom and surface depths of sampling in each site **(B)** and immersion design at each site **(C)**.

The five sites (i.e., 41p, Pt15, Pt12, MIS, and 6ext) of immersion were located throughout the semi-enclosed Toulon Bay (North-Western Mediterranean Sea, France), from the entrance of the bay to the north-western anthropized area (Figure 1). The five sites were chosen along multi-chemical contamination gradients, based on previous chemical characterization of the water column in Toulon Bay (Coclet et al., 2018).

Polycarbonate was chosen as a common plastic substrate found in seawater. PC plates were previously sandblasted to promote microbial adhesion. All plates have been immersed in the five sites at the surface (1 m depth) and the bottom (1 m above sediment) of the water column, over a period of one month, from June 1 to June 29, 2015. After 28 days (end of immersion), three biofilm samples, at each site and each depth were collected by scraping a 25 cm^2^ PC plate and washed with sterile artificial seawater. Biofilm samples were then placed upright in sterile cryotubes, and stored frozen at −80°C for later analysis (Briand et al., 2017). Additionally, a quantity of 1L seawater were collected from each site of immersion, at both surface and bottom of water column, every week over the one-month experiment, as described in Coclet et al. (2019). A total of 30 biofilm samples and 50 seawater samples were collected during the immersion experiment. Details of samples are shown in Supplementary Table 1.

### Environmental Measurements

Water temperature (°C), salinity, dissolved oxygen (mg L^–1^ and%) and chlorophyll *a* (μg L^–1^) were measured weekly at each site and depth using multi-parameter probe (Hydrolab DS5, OTT). Seawater samples for nutrients (DOC, NO_3_^–^, PO_4_^3–^) and dissolved TM were also collected weekly and analyzed as detailed in Coclet et al. (2018, 2019). Additionally, labile TMs concentrations, considered to be more representative from the bioavailable fraction (Caillat et al., 2014; Han et al., 2014; Kim et al., 2016), were assessed at every site and depth using three passive samplers (diffusive gradients in thin-films – DGT) immersed for the whole survey (*n* = 30). DGT devices, sample preparation and analytical methods for multi-element analysis in DGT eluates were described in Cindrić et al. (2017) and are summarized in Supplementary Material.

### DNA Extraction, 16S rRNA Gene Amplicon Sequencing

Biofilm materials were extracted using the PowerBiofilm DNA isolation Kit (Qiagen) following the supplier’s instructions, and DNA samples were conserved at −20°C. After DNA extraction, the V4–V5 region of 16S rRNA gene was amplified by PCR using 515F-Y/926R primer set (Parada et al., 2016). Details of PCR reaction and thermal cycling scheme is given in the Supplementary Materials. DNA extraction, amplification of seawater samples were fully described in Coclet et al. (2019) and briefly detailed in Supplementary Materials. Briefly, PCR products from all sampling dates were pooled for each site (*n* = 5) and each sampling depth (*n* = 2), in order to provide an overview of the bacterioplankton diversity during the survey and to compare with prokaryotic biofilm communities collected only once in each site. Amplicons were then paired-end sequenced (2 × 250 bp) with on an Illumina MiSeq platform at the GeT-PLaGe (Castanet-Tolosan, France).

### Bacterial Quantification

Quantification of 16S rRNA gene was performed by qPCR using primers BAC338f/515R (Borrel et al., 2012). Amplification reactions were performed in triplicate in a LightCycler 480 thermocycler (Qiagen) with GoTaq^®^ SybrGreen mastermix (Promega) in a final volume of 10 μL containing 0.25 μM of each primer and 2 ng of DNA. Serial 10-fold dilutions of a linearized recombinant plasmid ranging from 10^7^ to 10^2^ copies were also amplified in duplicate in each qPCR run to produce a standard curve used for determining 16rRNA gene copy number in the samples.

### Bioinformatic Analysis

Forward and reverse reads were merged using PEAR 0.9.8 with default options (Zhang et al., 2014a). Raw sequences were analyzed using MacQiime v.1.9.1 software (Caporaso et al., 2010). Briefly, barcode, primer, shorter sequences (<100 bp in length) and sequences with ambiguous base calls or homopolymer runs exceeding 10 bp were removed. The remaining sequences were clustered at a 97% threshold using Uclust algorithm (Edgar, 2010), including both closed and open reference operational taxonomic unit (OTU) picking, and taxonomy were assigned to each OTU by performing BLAST searches (Altschul et al., 1990) against the SILVA (release 128) database (Pruesse et al., 2007; Quast et al., 2013), with a maximum E-value of 1e–5. Low abundance OTU (<0.005%) were filtered as recommended by Bokulich et al. (2013). Sequences classified as mitochondria or chloroplast were removed from the OTU table, corresponding to 610 OTUs. A total of 233,120 reads were finally obtained representing 12,877 OTUs. OTU table was normalized by random subsampling to the smallest number of sequences (i.e., 5828). OUT table was also normalized by 16S rRNA gene copy number. Hierarchical clustering analysis using these data revealed that structure of biofilm communities differed by site but not by depth (Supplementary Figure 3A), due to the absence of significant difference between densities in surface and bottom biofilms. Thus, only qualitative data are used in the following manuscript. The 16S rRNA gene sequences have been deposited in the NCBI Sequence Read Archive (SRA) database under BioProject ID PRJNA522389.

### Functional Analysis

Functional profiles were predicted from obtained 16S rRNA gene data, using the R-based tool Tax4Fun2^1^ (Wemheuer et al., 2018), and based on KEGG category. The numeric results represent the fraction of the community that matched with the functional database, and indicated the community proportions containing each specific function. The functional predictions corresponded, on average to 17 ± 0.06% and 29 ± 0.19% of OTUs and sequences, respectively, according to the matches with the Tax4Fun2 reference functional dataset.

### Statistical Analysis

All statistical analysis and plots were under the R software (CRAN)^2^ and the GUI Rstudio using the core distribution (R Core Team, 2017). Details on data analysis is given in Supplementary Materials. Briefly, the alpha diversity was calculated in Qiime using Chao1, equitability, Observed OTUs, PD whole, Shannon and Simpson indices (Caporaso et al., 2010). Shapiro–Wilk and Bartlett’s tests were employed to test the data normality and homoscedasticity prior to other statistical analysis. All the tested variables followed parametric test conditions. One-way and two-way ANOVA tests and *post hoc* comparisons were used to assess differences between lifestyle, sampling sites and depths for labile metal concentrations, alpha diversity indices and prokaryote densities. Principal component analysis (PCA) was employed to observe differences in environmental profiles between both sampling sites and depths, using *FactoMineR* package (Lê et al., 2008). Only significant environmental variables (*p* < 0.01) were fitted on the PCA plot using envfit function (*vegan* package) (Oksanen et al., 2016). Non-metric multidimensional scaling (nMDS) ordination and Hierarchical clustering analysis based on Bray-Curtis dissimilarity matrices, using the *vegan* package, was used to visualize taxonomic composition profiles. Permutational multiple analysis of variance (PERMANOVA) tests were used to identify significant differences in bacterial community structure between lifestyle, sampling sites, and depths. To investigate the relationships between both biofilm structure and functions and environmental variables, redundancy analysis (RDA) was performed on the global community with *vegan* package in R, using Hellinger transformed data and a reduced and normalized environmental dataset, as already described (Coclet et al., 2018, 2019). Finally, we used Welch’s tests to detect significant taxonomic composition and functional differences between the communities (biofilm vs seawater; Surface vs Bottom; Contaminated vs Uncontaminated), using STAMP (Statistical Analysis of Metagenomic Profiles) software v 2.1.3 (Parks and Beiko, 2010). All parameters and outputs of statistical tests performed (ANOVA and PERMANOVA) were provided in Supplementary Tables S3 **to** S5.

## Results

### Physico-chemical Variability in Seawater

**FIGURE 2:**
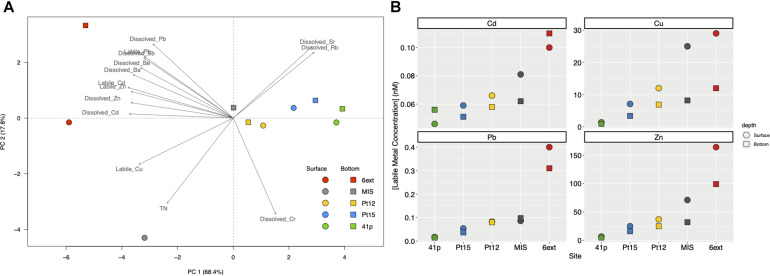
Principal component analysis (PCA) biplot based on the environmental variables of surface and bottom seawater samples collected at the five different sites **(A)** and labile trace metals concentrations (Cd, Cu, Pb, and Zn) in surface and bottom of seawater at the different sampling sites. **(B)** Dotplots represent the labile metal concentrations (in nM), along each sampling sites (i.e. 6ext, MIS, Pt12, Pt15, and 41p) in surface biofilm (circle) and bottom biofilm (square) samples. Colors and shapes of symbols depend on site sampling and depth sampling, respectively.

Thirty-three physicochemical variables were measured in the Toulon Bay seawater, used to performed the PCA (Figure 2A), and presented in Figure 2B and Supplementary Table 2. Among these 33 environmental variables, 14 were significantly discriminants (envfit, *p* < 0.01), and explained 86.2% of the total variation of the environmental dataset. The main contributors were TM concentrations (e.g., dissolved and labile Cu, Cd, Pb and Zn) for PC1 (68.4%), and total nitrogen, and dissolved Cr for PC2 (17.8%). Environmental variables differed significantly between sites (PERMANOVA, *p* < 0.01) but not between depths (PERMANOVA, *p* > 0.1).

Among significant variables, both dissolved and labile Cd, Cu, Pb, and Zn concentrations increased from the uncontaminated site (41p) to the most anthropized sites (MIS and 6ext) (ANOVA; *p* < 0.05) (Figure 2B and Supplementary Table 2). Concentrations of labile Cd in the most anthropized sites (0.1 nM) were up to 2-fold higher than those observed in the uncontaminated sites (0.046 nM). Concentrations of labile Cu in the most anthropized sites (29 nM) were up to 31-fold higher than those observed in the uncontaminated sites (0.93 nM). Concentrations of labile Pb in the most anthropized sites (0.4 nM) were up to 20-fold higher than those observed in the uncontaminated sites (0.02 nM). Concentrations of labile Zn in the most anthropized sites (164 nM) were up to 35-fold higher than those observed in the uncontaminated sites (4.9 nM). Finally, there was no significant difference of both dissolved and labile TM concentrations between surface and bottom seawater.

### Alpha Diversity and Density of the Prokaryotic Communities

**FIGURE 3:**
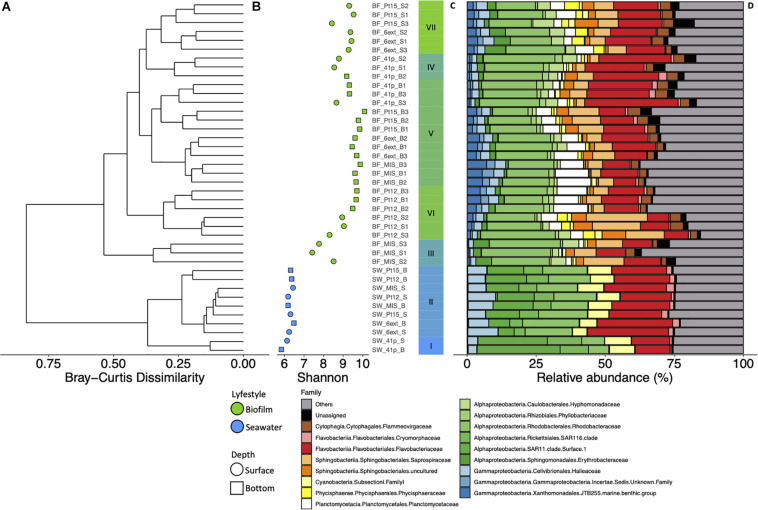
Dendrogram clustering represents the Bray-Curtis dissimilarity of taxonomic profile, conducted from the OTU abundance table **(A)**. The scale bar of the dendrogram represents the dissimilarity level (%) between prokaryotic community. Shannon index values calculated from 16S rRNA OTU table along each sampling sites (i.e., 6ext, MIS, Pt12, Pt15 and 41p) in surface (circle) and bottom (square) samples for biofilm (green) and seawater (blue) samples **(B)**. Barplot representing the cluster from the Bray-Curtis dendrogram **(C)**. Structure of prokaryotic community at the family level in seawater and biofilm at surface and bottom waters at the five different sites of Toulon Bay **(D)**. Each bar represents the relative abundance of family in the studied samples.

All α-diversity indexes differed significantly between depths (ANOVA; *p* < 0.001) and sites (ANOVA, *p* < 0.05) (Figure 3B and Supplementary Figure 1). Shannon index was significantly higher in bottom biofilms (BB) than in surface biofilms (Figure 3B). Lowest values of Shannon index were found at MIS while Pt15 and 6ext sites displayed the highest values of Shannon index. Finally, independently of the site and depth, richness and diversity indices were significantly higher in biofilm than in seawater samples (Figure 3B and Supplementary Figure 6) (ANOVA; *p* < 0.05).

Prokaryotic densities into biofilms significantly differed between sites (ANOVA, *p* < 0.001) while no difference between depths was observed (ANOVA, *p* > 0.05). The highest and lowest estimated densities were observed in Pt15 (1.2 ± 0.2 × 10^7^ cell mL^–1^) and MIS (6.6 ± 2.0 × 10^5^ cell mL ^–1^), respectively (Supplementary Figure 2).

### Structure of Prokaryotic Communities Between Surface and Bottom Biofilms

Hierarchical clustering analysis firstly revealed that structure of prokaryotic SB (i.e., clusters III, IV, VII) significantly differed from those of BB (i.e., cluster V) communities (PERMANOVA, *p* < 0.001) (Figure 3B and Supplementary Figure 3B). Among the different phyla, Proteobacteria (49% of total reads), and Bacteroidetes (31% of total reads) were the most abundant in all biofilm communities (i.e., clusters III–VII) (Figures 3C,D). At the family level, prokaryotic biofilm communities were dominated by *Rhodobacteraceae* (19 ± 0.68%), *Flavobacteriaceae* (15 ± 1.1%), *Saprospiraceae* (7.7 ± 0.80%), *Planctomycetaceae* (5.4 ± 0.62%), *Flammeovirgaceae* (3.2 ± 0.17%), and *Hyphomonadaceae* (3.2 ± 0.33%).

Based on STAMP results, 22 families have been identified to show a significant difference in relative abundance between SB and BB communities (Welch’s test; *p* < 0.05; Effect size < 0.2) (Supplementary Figure 4). *Hyphomonadaceae* (4.6 ± 1.6%), *Erythrobacteraceae* (3.5 ± 2.1%), *Phycisphaeraceae* (2.6 ± 1.7%), *Parvularculaceae* (1.2 ± 0.91%), and unknown *Alphaproteobacteria* (0.93 ± 1.7%) families were significantly more represented in SB than in BB communities. Among the 17 families significantly more represented in BB than in SB communities, *Planctomycetaceae* (7.3 ± 3.5%), *JTB255 marine benthic group* (MBT) (3.6 ± 1.8%), *Sva0996 marine group* (1.2 ± 0.34%), unknown *Gammaproteobacteria* (1.9 ± 0.92%), *Desulfobulbaceae* (0.29 ± 0.21%), *Phyllobacteriaceae* (1.8 ± 0.44%), *Hyphomicrobiaceae* (1.1 ± 0.98%), and *Rhodobiaceae* (1.1 ± 0.42%) exhibited the higher difference.

**FIGURE 4:**
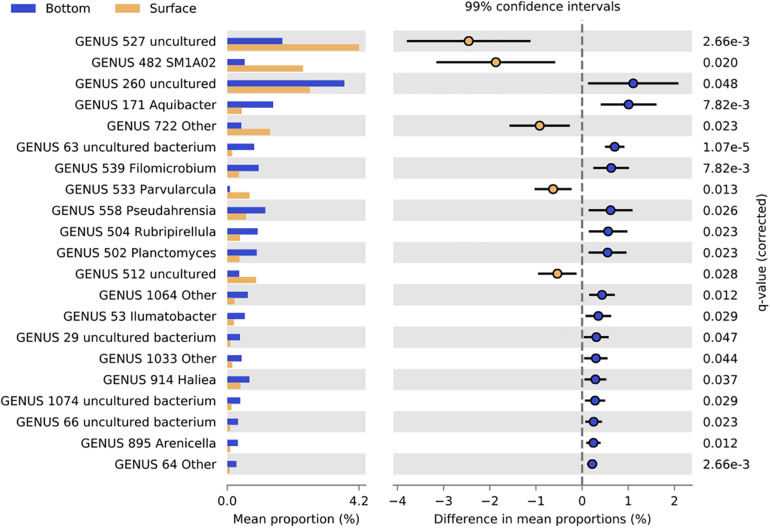
Bar graph representing relative proportions of significant genera in biofilms from surface (yellow) and bottom (blue) samples. Extended error bar plots showed pairwise comparison of significant genera proportions (Welch’s *t*-test; *p* < 0.01) between surface (yellow) and bottom (blue) samples. Corrected *p*-value is determined using Fisher’s exact test.

At the genus level, 21 genera showed significant differences in abundance between SB and BB communities (Welch’s test; *p* < 0.05; Effect size < 0.2) (Figure 4). Uncultured *Hyphomonadaceae*, *Phycisphaeraceae* SM1A02, *Erythrobacteraceae* Other, *Parvularcula*, and uncultured *Alphaproteobacteria* genera were significantly more represented in SB than in BB communities. Finally, among the 16 genera which were significantly more represented in BB than in SB communities, uncultured *Saprospiraceae*, *Aquibacter*, uncultured bacterium *Sva0996 marine group*, *Filomicrobium*, *Pseudahrensia*, *Rubripirellula*, and *Planctomyces* exhibited the higher difference in mean proportions between SB and BB.

### Structure of Prokaryotic Communities in Biofilm Between Immersion Sites

In addition to the depth structuration, the hierarchical analysis revealed secondly that structure of prokaryotic biofilm communities differed significantly between sites (PERMANOVA, *p* < 0.001) (Figure 3 and Supplementary Figure 3B). STAMP analyses revealed that seven families exhibited significant difference in abundance between sites in SB communities (Welch’s test; *p* < 0.01, Effect size < 0.9) (Supplementary Figure 5A). In BB communities, 15 families exhibited significant difference in abundance between sites (Welch’s test; *p* < 0.01, Effect size < 0.9) (Supplementary Figure 5B). Except *JTB255 MBG, Phyllobacteriaceae*, and *Hyphomicrobiaceae*, all discriminant families represented less than 1% of the total bacterial community.

### Characterization of Prokaryotic Communities in Biofilms and Seawater

**FIGURE 5:**
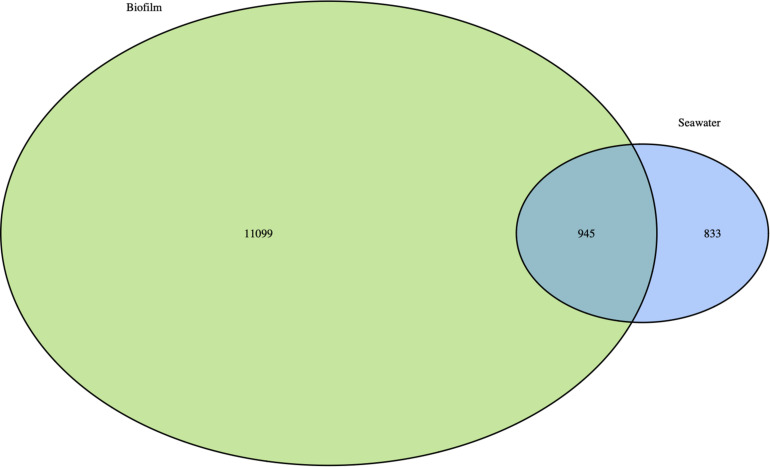
Venn diagram showing prokaryotic OTUs overlap for pooled biofilm samples, and seawater samples at Toulon Bay. Numbers inside the circles represent the number of shared or unique OTUs for the given environment.

Prokaryotic communities of biofilms and seawater were dominated by members of Proteobacteria which represented 58 and 49%, and Bacteroidetes which represented 27 and 31% of total reads, respectively. However, substantial differences in the structure between biofilm and planktonic communities were observed at the phylum level and below (Figures 3A–D). UPGMA clustering showed that bacterioplankton (clusters I and II) and biofilm (clusters III to VII) communities were clearly distinct, showing only 25% of Bray-Curtis similarity (PERMANOVA, *R*^2^ = 0.40 to 0.65, *p* < 0.001) (Figure 3A). The distinctness of prokaryotic communities was also reflected in the number of shared OTUs across the two lifestyles, with 945 OTUs (7.3%) shared between the biofilm and seawater samples (Figure 5). Biofilm had the largest number of unique OTUs (only observed in this compartment) (*n* = 11099; 86%), mostly rare OTUs (<1% of all sequences across all samples). Bacterioplankton had the lowest number of unique OTUs (*n* = 833; 6.5%).

**FIGURE 6:**
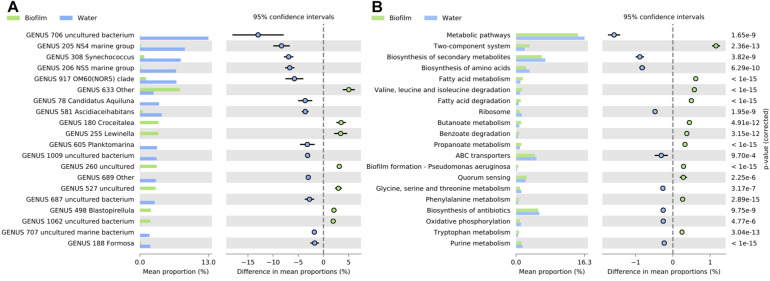
Bar graph representing relative proportions of significant genera **(A)** and KEGG Pathways at level 3 of SEED metabolic hierarchy **(B)** in biofilms (green) and seawater (blue) samples. Extended error bar plots showed pairwise comparison of significant genera **(A)** and KEGG Pathways at level 3 of SEED metabolic hierarchy **(B)** proportions (Welch’s *t*-test; *p* < 0.01) between in biofilms (green) and seawater (blue) samples. Corrected *p*-value is determined using Fisher’s exact test.

STAMP analyses revealed that 21 families and 37 genera exhibited significant difference in abundance between biofilm and bacterioplankton communities (Welch’s test; *p* < 0.01, Effect size < 1) (Figure 6A and Supplementary Figure 7). At the genus level, among the 16 genera which were significantly more represented in bacterioplankton than in biofilm communities, uncultured *SAR11 clade Surface 1*, *NS4 marine group*, *Synechococcus*, *NS5 marine group*, *OM60(NOR5) clade*, *Candidatus Aquilina*, *Ascidiaceihabitans*, and *Planktomarina* genera exhibited the higher difference in mean proportions. Conversely, *Rhodobacterales* Other, *Croceitalea*, and *Lewinella*, uncultured *Saprospiraceae*, and uncultured *Hyphomonadaceae* genera were significantly more represented in biofilms than in bacterioplankton communities.

### Predicted Functions of Prokaryotic Communities in Both Biofilms and Seawater

A total of 246 KEGGs (functional orthologs) within 27 metabolic pathways were identified and used for the global functional analysis. Firstly, predicted function profiles between biofilm and seawater samples were significantly different (PERMANOVA, *p* < 0.001). Additionally, sites (PERMANOVA, *p* < 0.001), mainly, and depths (PERMANOVA, *p* < 0.002), significantly discriminated the predicted functional profiles of biofilm samples (Supplementary Figure 3C).

Globally, biofilms were enriched with KEGG pathways related to Xenobiotics biodegradation and metabolism, lipid metabolism, signal transduction, and cellular community functions, and specifically by the two-components system, fatty acid metabolism and degradation, Valine, leucine and isoleucine degradation, biofilm formation and quorum sensing functions (Welch’s test; *p* < 0.01, Effect size < 0.01) (Figure 6B and Supplementary Figure 8). On the contrary, seawater samples were significantly enriched with KEGG pathways related to global and overview maps, energy metabolism, translation, and nucleotide metabolism, mainly with biosynthesis of secondary metabolites, biosynthesis of amino acids, and ABC transporters’ functions (Welch’s test; *p* < 0.01, Effect size < 0.01) (Figure 6B and Supplementary Figure 8).

**FIGURE 7:**
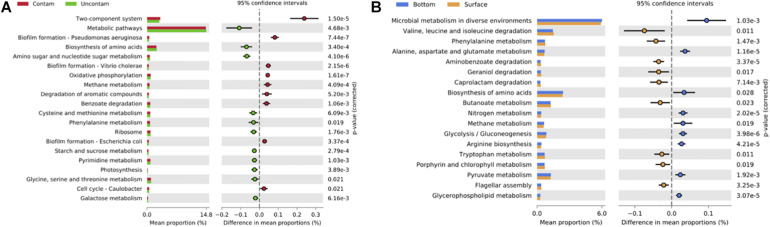
Bar graph representing relative proportions of significant KEGG Pathways at level 3 of SEED metabolic hierarchy in biofilms from contaminated (6ext; red) and uncontaminated (41p; green) sites **(A)**, and in biofilms from surface (yellow) and bottom (blue) **(B)**. Extended error bar plots showed pairwise comparison of significant KEGG Pathway proportions (Welch’s *t*-test; *p* < 0.01) at level 3 of SEED metabolic hierarchy between biofilms from contaminated (6ext; red) and uncontaminated (41p; green) sites **(A)**, and between biofilms from surface (yellow) and bottom (blue) **(B)**. Corrected *p*-value is determined using Fisher’s exact test.

*Post hoc* tests revealed also that main differences in functional profiles were between biofilms from the most and less contaminated sites, 6ext and 41p, respectively. The most contaminated site was enriched with KEGG pathways that were related to “Signal transduction” and “Xenobiotics biodegradation and metabolism”, and more specifically to two components system, biofilm formation, oxidative phosphorylation, methane metabolism, degradation of aromatic compounds, and benzoate degradation functions (Welch’s test; *p* < 0.01, Effect size < 0.01) (Figure 7A and Supplementary Figure 9). On the contrary, the uncontaminated site was enriched with KEGG pathways that were related to “Amino acid and carbohydrate metabolisms”, and more precisely to metabolic pathways, biosynthesis of amino acids, amino sugar and nucleotide sugar, cysteine, methionine, and phenylalanine metabolisms’ functions (Welch’s test; *p* < 0.01, Effect size < 0.01) (Figures 7A,B and Supplementary Figure 9).

### Relationships Between Prokaryotic Structure or Predicted Functions in Biofilm Communities and Environmental Variables

**FIGURE 8:**
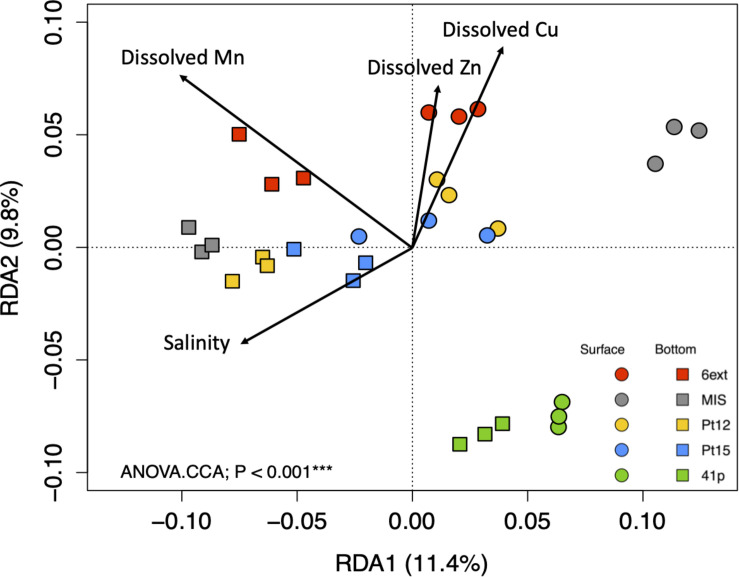
Redundancy analysis (RDA) ordination diagram of the first two axes for KEGG Pathway profiles at level 3 of SEED metabolic hierarchy. The percentage of the spatial variation in community structure explained by each axis is indicated in parentheses after the axis label. The constrained sets of environmental variables analyzed are indicated as vectors. *P*-value correspond to the results of CCA.ANOVA analysis.

The contribution of measured environmental variables in the variations of biofilm community structure and function was tested by RDA analyses. While, SB and BB samples formed two separate clusters, total set of environmental variables didn’t contribute significantly to the variation in the biofilm community structure (ANOVA.CCA, *p* > 0.05). On the contrary, according to the RDA analysis, a significant difference was found in biofilm community function profiles between samples (ANOVA.CCA, *p* < 0.001), mainly explained by dissolved Cu, Zn, Mn, and salinity (Figure 8). In accordance to nMDS (Supplementary Figure 3C), functional profiles from biofilm samples showed separation between sites and depths, with the 4 significant environmental variables, explaining 20% (for the first two RDA axes) of the function variability. RDA analysis clearly identified TM (Cu, Zn, and Mn), as the dominant abiotic drivers of predicted function profiles.

## Discussion

By combining measurement of physicochemical parameters with variations in prokaryotic biofilm diversity and functional potential, we provided a first insight into the marine prokaryotic biofilm ecology of a highly anthropized coastal area, such as the Toulon Bay. Overall, abiotic variables were stable through the one-month experiment in June 2015, and no significant differences between surface and bottom seawater were observed. However, we found that TM concentrations exhibited strong north-south decreasing gradients, as previously observed punctually, monthly, or pluri-annually in Toulon Bay (Coclet et al., 2018, 2019, 2020; Layglon et al., 2020), allowing us to specifically address the potential effect of TM on the structure and functions of prokaryotic biofilm communities. The high contamination levels of localized areas indicated significant anthropogenic inputs that could be attributed to numerous recent (e.g., large boat traffic, harbor activities, antifouling coatings, sediment remobilization) (Turner, 2010; Layglon et al., 2020), and historical events (2nd World War) (Tessier et al., 2011).

### Limited Impact of Trace Metal Gradients on Prokaryotic Biofilm Structure

While we observed a significant impact of the site of immersion on prokaryotic biofilm structure, and despite the uncontaminated site 41p appeared as a separate cluster, surprisingly this was not related to TM contamination gradients or to other measured environmental variables in this study. Conversely to bacterioplankton communities (Coclet et al., 2018, 2019), biofilm communities could exhibit better adaptation and survival skills, as they are protected within a matrix of EPS and show ability to immobilize the pollutants (Mohapatra et al., 2020). Indeed, complex natural biofilms include a diversity of organisms with different metabolic capacities and physiologies which generates opportunities for cooperation (Dang and Lovell, 2016; Flemming et al., 2016; Kirstein et al., 2018) and promote tolerance (Königs et al., 2015) or resistance to metallic stress (Harrison et al., 2007). Interestingly, biofilms respond dynamically to pollutant substances, suggesting that biofilms can be viewed as fortresses made of a self-produced matrix of EPS (Flemming and Wingender, 2010; Flemming et al., 2016). Intuitively, the matrix of EPS might plausibly represent a diffusion barrier. EPS components of the matrix can substantially annihilate the activity of toxic substances that diffuse through the biofilm (Billings et al., 2015). For example, in response to the exposure to toluene, biofilm was reported to produce EPS enriched in carboxyl groups which can lead to an increase in their ions exchange capacity (Harrison et al., 2007; Flemming et al., 2016). The matrix of biofilms can also accumulate metals such as Cu^2+^, Zn^2+^, Fe^2+/3+^, and Al^3+^, which protects the biofilm from the toxicity of the metal ions (Harrison et al., 2007; Grumbein et al., 2014; Flemming et al., 2016; McElroy et al., 2016), especially when present at concentrations toxic to free-living cells like observed in Toulon Bay (Coclet et al., 2018). Copper can be complexed by polysaccharides in the EPS matrix in order to protect the biofilm community (Ordax et al., 2010). Additionally, numerous mechanisms of detoxification in biofilm, including metabolic heterogeneity, extracellular signaling, metal immobilization and complexing, and uptake of resistance genes by HGT are well known and described in the literature (Decho, 2000; Teitzel and Parsek, 2003; Harrison et al., 2007; Mah, 2012). However, future studies are needed to specify the response of prokaryotic biofilms to specific contamination, such as Cu or Pb alone.

The established inherent capacity of biofilms to survive in contaminated environments is also explained by their ability to play a role in bioremediation processes (Singh et al., 2006; Mohapatra et al., 2020). Biofilms are more suitable for the remediation of TM compounds because of their high microbial cell densities, structural stability, signaling process, metabolic diversification, immobilization ability, and the presence of surface active molecules (Singh et al., 2006). Biofilm can efficiently remove metals by the bioaccumulation and biosorption mechanisms due to their high biomass density and can also reduce some metals to a lower toxicity level by their enzyme activities (Harrison et al., 2007; Flemming and Wingender, 2010).

The susceptibility and the capacity of resistance to multi-metal contamination, as well as the ability to play a role in bioremediation and immobilization of TM depends on the nature of the biofilm microorganisms (Harrison et al., 2007). The presence of *JTB255 MBG, Alcanivoracaceae*, and *Porticoccaceae* (Gammaproteobacteria), Hyphomicrobiaceae (Actinobacteria), and Methanobacteriaceae (Euryarchaeota) families, as key taxa in biofilms from the most contaminated site of Toulon Bay is relevant with the literature, in the sense all these taxa have already been observed in harsh-condition environments, as metal-contaminated seawaters, sediments and biofilms (Sjöstedt et al., 2012; Bell et al., 2013; Emilson, 2015; Liu et al., 2015; Mußmann et al., 2017; Won et al., 2017; Garris et al., 2018; Yan et al., 2018; Yakimov et al., 2019; Knapik et al., 2020). Most of these groups have been identified as tolerant to contaminants in biofilm communities (Yang et al., 2016; Pollet et al., 2018; Catão et al., 2019). The *Alcanivoracaceae* family contains a large group of hydrocarbon-degrading bacteria, due to their ability to use hydrocarbons as main carbon source (Yakimov et al., 2019). Most family members, such as *Alcanivorax* and *Ketobacter*, are highly specialized in degrading linear and branched alkanes of different origin. They typically dominate marine environments suffering from oil contamination (Won et al., 2017). They are also known to form biofilms around oil droplets and at the oil–water interface (Coulon et al., 2012). Even if the ability to degrade hydrocarbons is widespread among marine prokaryotes, the majority of specialized hydrocarbon-degrading microorganisms belong to Gammaproteobacteria (Coulon et al., 2012; Yakimov et al., 2019), such as *Alcanivoracaceae*, or *JTB255 MBG* and *Porticoccaceae* found in this study. Finally, *Hyphomicrobiaceae* were previously identified in disturbed environments, suggesting that they could be promising bioindicators for monitoring the impact of contamination (Sjöstedt et al., 2012; Simonin et al., 2019). Taken together, the presence of these key taxa in prokaryotic biofilms from contaminated sites could explain the limited impact of metals on the biofilm structure along TM contamination gradients of Toulon Bay. This suggest that marine prokaryotic biofilms may be able to survive to strong metal contamination because of the high diversity of the biofilm community, and the nature of the population structure, as well.

Finally, among not measured environmental factors that could be involved in the driving of biofilm communities, organic matter availability and nature would be relevant to consider, as already proposed for bacterioplankton communities in the bay of Toulon (Coclet et al., 2019). It makes sense considering that organic matter, in addition to determine the source of carbon and nutrients for all the microbes, probably influences the biochemical surface conditioning, that come before the colonization process (Flemming and Wingender, 2010).

### Trace Metal Contamination Impacts Predicted Functional Profiles of Biofilms

In contrast to the structure of biofilm communities, our results indicated that predicted functional biofilm profiles could be explained by some of the environmental variables we measured., Indeed, TM contamination, especially Cu, Mn, and Zn, seemed to be the main driver of predicted functional profiles of biofilm communities. Pathways involved in Amino acid and Carbohydrate metabolisms, such as metabolic pathways, biosynthesis of amino acids, amino sugar and nucleotide sugar, cysteine, methionine, and phenylalanine metabolisms dominated the functional profiles of the overall biofilm communities, but they were mainly represented in biofilms from the uncontaminated site. The dominance of the functions related to maintenance of basic cellular machinery, enabling growth and metabolism was consistent with the results from previous comparative metatranscriptomic analyses of disturbed aquatic (Parro et al., 2007; Moreno-Paz et al., 2010; Bertin et al., 2011; Bergsveinson et al., 2020) and marine environments (Chen et al., 2015b; Ding et al., 2019), indicating that the biofilm community is capable of maintaining normal growth processes and metabolic functions in face of metal toxicity, such as biofilms from uncontaminated environments.

On the contrary, pathways involved in Xenobiotics biodegradation and metabolism, such as methane metabolism, degradation of aromatic compounds, and benzoate degradation functions dominated the functional profiles of biofilm communities from the contaminated sites. Over-representation of metabolic pathways involved in the degradation of aromatic compounds (hydrocarbons) and xenobiotics, and degradation of toxic compounds observed in biofilms from the contaminated sites, provide further evidence for the influence of anthropogenic activity on prokaryotic function. These results are relevant with those found in several studies (Ding et al., 2019; Bergsveinson et al., 2020), where these functions have been found in biofilms submitted to Zn exposure. As expected, the predicted functions enriched in overall biofilms were related to ion transport, ion resistance, prokaryotic defense, and DNA transfer. This result suggests that functions associated with TM transport and resistance should be over-expressed in biofilm communities from contaminated environments in the Toulon Bay. These findings can imply that one important feature for the biofilm communities developed in highly contaminated environment is the occurrence of metal ion transport and resistance genes in their genomes.

Biofilms from the contaminated sites also harbored higher KEGG functions for two-components system, involved in quorum sensing signaling and EPS matrix formation, as well as biofilm formation (Lee et al., 2009; Yelton et al., 2013; Chen et al., 2015a). Both two-components signal transduction and biofilm formation systems enable bacteria to sense, respond, and adapt to changes in their environment or in their intracellular state, by participating to biofilm formation (i.e., Quorum sensing pathway) and EPS matrix production (i.e., peptidoglycan biosynthesis and extracellular polysaccharide production, and adhesion genes). The two-components system contains also genes associated with membrane transport, and in particular, multiple efflux systems. While these pump systems also have role in antibiotic resistance and potential xenobiotic degradation, the preference for these transport systems over the minimally ABC transporters in biofilms from the contaminated sites, indicates that the hydrolysis of ATP to drive ABC transport is not an optimal energy expenditure for biofilms exposed to metal contamination (Parro et al., 2007; Moreno-Paz et al., 2010; Bergsveinson et al., 2020).

Despite Tax4Fun2 provides a good approximation to functional profiles obtained from metagenomic shotgun sequencing approaches, relatively low fraction of reads (approximatively 30%), classified by QIIME, were used to predict the functional profiles, likely caused to the high complexity of the natural marine biofilm communities, which are poorly represented in the KEGG database (Wemheuer et al., 2018). Moreover, although the KEGG Orthology database evolved rapidly with new functional orthologs, predicted functions were mostly affiliated to human microbiome. Predicted functions related to both metal tolerance and resistance, metal stress response, as well as, to metal acquisition processes such as membrane transport are not still very available in the KEGG database. It must be noted that the functional predictions cannot replace whole metagenome shotgun sequencing approach. Such analyses would provide useful insights of metabolic activities and functional profiles of microorganisms into prokaryotic biofilms submitted to harsh conditions. However, the results of our study can serve as template for further metagenomic or metatranscriptomic studies on marine microbial biofilm communities in a highly contaminated area.

### Prokaryotic Structure Exhibited Variations Between Surface and Bottom Biofilms

In this study, we also tried to improve our understanding of spatial variations of prokaryotic biofilms’ community structure through the comparison of surface and bottom water column in shallow coastal areas (i.e., 10-m depth on average). Very scarce data exist in the literature as depth impacts on biofilm community on artificial substrate was only studied in extreme oligotrophic conditions in the deepest part of the Mediterranean Sea between 1,500 and 4,500 m depth (Bellou et al., 2012, 2020). Our results showed that, whatever the site, the structure of biofilm communities formed on immersed PC plates at the surface was dissimilar to bottom ones. Then, conversely to bacterioplankton community (Coclet et al., 2019), the structure of biofilm communities seemed to exhibit a vertical structuration along the seawater column. However, as shown by the RDA analysis, this spatial structuration seemed to be driven by other environmental factors than the variables measured in this study. The analysis of the abiotic variables showed that the geochemical profile was homogeneous from the surface to the bottom waters, probably because of the low depth. The influence of light attenuation with depth in such rather turbid environments, not measured here, remains to be further appreciated.

The sediment of the Toulon Bay is also known for its organic contamination by PAHs and PCBs (not measured in this study), the contamination of these compounds increasing globally from South-East to North-West as for TMs (Wafo et al., 2016). Moreover, resuspension experiments have demonstrated that organic matter and PAH could efficiently be remobilized into the water column (Guigue et al., 2017). Considering that remobilized organic matter and PAH can sustain the growth of specific bacterial lineages (Quero et al., 2015; Jeanbille et al., 2016; Salerno et al., 2018), multi-contaminated areas of Toulon Bay could provide higher diverse microbial communities than marine uncontaminated coastal areas. The presence of known hydrocarbonoclastic taxa in this study, including Gammaproteobacteria, such as *Alcanivoracaceae*, or *JTB255 MBG* and *Porticoccaceae* tends to confirm the influence of organic contaminants hypothesis (Coulon et al., 2012; Dussud et al., 2018; Yakimov et al., 2019). This hypothesis is also supported by our observation of a higher diversity in BB samples which is consistent with similar conclusions proposed for bacteria within the sediments of Toulon Bay (Misson et al., 2016). Webster and Negri (2006) also suggested that organic (PAHs) pollution levels in sediments may have a direct impact on the community structure of microbial biofilms.

Members of *Planctomycetaceae* (*Plnactomyces*), *JTB255 MBG, Desulfobulbaceae, Phyllobacteriaceae*, and *Rhodobiaceae* significantly discriminated BB’s communities from SB ones. Most of these groups were also observed in contaminated sediments (Webster and Negri, 2006; Zhang et al., 2008; Besaury et al., 2012; Acosta-González et al., 2013; Fonti et al., 2014; Quero et al., 2015; Wang et al., 2016; Fang et al., 2017; Won et al., 2017; Dai et al., 2018; Godoy-Lozano et al., 2018; Miao et al., 2019; Song et al., 2019; Yakimov et al., 2019). The presence of Planctomycetes as biomarkers of bottom biofilms in our study is consistent with the results obtained in Antarctic marine biofilms established on glass surfaces near highly contaminated sediments (Webster and Negri, 2006). Taken together, our results suggest that surrounding superficial sediments could possibly serve as a direct source for the colonization of nearby immersed substrates by opportunistic microorganisms at the sediment/water interface, as already reported for freshwater ecosystems (Battin et al., 2001). This process could happen with sediment resuspension during storm events or human activities, such as dredging operations, which are numerous in Toulon Bay (Layglon et al., 2020). Investigating the benthic compartment of the Toulon Bay appears necessary to shed the light on the role of both benthic communities and sediment contamination on biofilm formation and establishment in a multi-contaminated ecosystem.

### Structure and Predicted Functions Are Dissimilar Between Biofilm and Planktonic Communities

In this study, by analyzing 16S rRNA gene amplicon sequences from biofilms and surrounding seawaters, we comprehensively explored both structure and functions related to both prokaryotic biofilms and planktonic communities in the highly contaminated Toulon Bay. Alpha diversity showed a clear distinction between lifestyles with higher diversity in biofilms. The higher diversity in biofilm samples could be likely related to an addition of rare planktonic OTUs, undetectable in seawater and variable in time, which are able to colonize and proliferate into biofilms. These findings support several previous studies pointing toward a consensus that bacterioplankton community structure differs from immersed artificial surface attached ones (Zettler et al., 2013; Oberbeckmann et al., 2014, 2016; Amaral-Zettler et al., 2015; De Tender et al., 2015, 2017; Bryant et al., 2016; Kirstein et al., 2018; Ogonowski et al., 2018; Ding et al., 2019), including in the Toulon Bay (Catão et al., 2019), by selecting rare and/or specialists into a pool of bacterioplankton community.

Additionally, the beta-diversity analysis suggests that the variation in sampling sites has lower effect than depths on the taxonomic composition of biofilms, whereas niche differentiation between biofilms and planktonic communities may play an essential role in determining the microbial community structure. Although prokaryotic planktonic and biofilm communities possessed common taxonomic groups considering the overall community structure, differences of structure between both lifestyles were observed from phylum to species, and a huge number of taxa were only detected in the biofilms. Taxonomic comparison between biofilm and seawater suggests that species sorting, referring to selection from the pool of microbes in surrounding seawater, may play important roles during biofilm formation (Langenheder and Székely, 2011; Meier et al., 2013; Ding et al., 2019). Plastic, whatever its nature (Kirstein et al., 2018), is known to provide a novel habitat for microorganisms and that species sorting occurs, particularly, during the early colonization stages (Harrison et al., 2007; Zhang et al., 2014b; Bryant et al., 2016; Ogonowski et al., 2018; Pollet et al., 2018). Pioneer stages were restricted to specialists displaying adhesion capacity and/or exopolymeric secretion potential and probably communication ability (McDougald et al., 2011; Flemming et al., 2016).

The increase in diversity over time, leading to highly diversified biofilms as reported here, was associated to the significant changes between predicted functional profiles from biofilm and planktonic communities. Most of the functions related to biofilm formation and compound degradation were enriched for biofilms, whereas functions involved in biosynthesis of secondary metabolites, biosynthesis of amino acids, and ABC transporters were prominent in bacterioplankton communities. These observations tend to confirm the better capacities of defense of biofilm communities against metal contamination, compared to surrounding bacterioplankton communities, as already shown (Moreno-Paz et al., 2010; Wei et al., 2013; Ding et al., 2019).

Our results are in agreement with the common observation of major differences between both structure and function profiles of marine biofilms and of surrounding bacterioplankton. These results reinforce the hypothesis of an increase in diversity into biofilms due to the recruitment of generalists, a diversification of the structure between biofilm and planktonic communities, and accurate defense mechanisms against metal contamination by biofilm communities through the functional response, as well.

## Conclusion

Our study provides one of the first detailed description of natural and complex prokaryotic biofilm communities in a highly anthropized marine area. The analysis of 16S rRNA gene amplicon sequences revealed that TM contamination in Toulon Bay have higher impacts on the predicted functional profiles than on the structure of biofilm communities. The structure of biofilm communities was stable along TM contamination gradients, with however, the presence of specialized hydrocarbon-degrading microorganisms or resistant taxa, known to play a role in bioremediation and immobilization of metals. Most of functions found in biofilms from the contaminated site were closely related to metal ion transport, resistance genes, and alteration to membrane integrity, as well as biofilm formation mechanisms. Taken together, our results suggest that biofilms are less impacted than planktonic communities to metal contamination, due to a protection offered by the resistant species to all members in the community, and a wide range of specific mechanism of protection. In addition to metal contamination, our results showed that benthic communities or environmental variables into sediments could influence the structure and the functional profiles of prokaryotic biofilm communities, due to sediment remobilization processes. With the benefit of ‘omics’ approaches, such as the coupling of metagenomics and metatranscriptomic analyses, future studies could be designed to explore gene transcription profiles along TM contamination gradients for both biofilm and planktonic communities. Future studies are also needed to bring new evidences about the role of sediments in biofilm colonization and their impact on the structure and functions of biofilm communities.

## Data Availability Statement

The datasets presented in this study can be found in online repositories. The names of the repository/repositories and accession number(s) can be found in the article/Supplementary Material.

## Author Contributions

CG, J-FB, and BM proposed and designed the study. CC, CG, GD, SD, CL, DO, J-UM, J-FB, and BM organized and performed field sampling. CC, CG, GD, DO, CL, DE, SD, and BM analyzed the samples. CC, CG, J-FB, and BM interpreted the results and participated in manuscript redaction. All authors approved publication of the content.

## Conflict of Interest

The authors declare that the research was conducted in the absence of any commercial or financial relationships that could be construed as a potential conflict of interest.

## Footnotes

1 https://sourceforge.net/projects/tax4fun2/

2 http://cran.r-project.org/
