# Supplementary material for: Trace Metal Contamination Impacts Predicted Functions More Than Structure of Marine Prokaryotic Biofilm Communities in an Anthropized Coastal Area
Source: Front Microbiol. 2021 Feb 19;12:589948. doi: 10.3389/fmicb.2021.589948 (PMC7933014; doi:10.3389/fmicb.2021.589948)
Supplement: Supplementary file 1 [file Data_Sheet_1.docx]

***Supplementary Information***

**Trace metal contamination impacts predicted functions more than structure of marine prokaryotic biofilm communities in an anthropized coastal area**

Clément Coclet^a,b,1,^, Cédric Garnier^b^, Sébastien D’Onofrio^b^, Gaël Durrieu^b^, Emilie Pasero^c^, Christophe Le Poupon^b^, Dario Omanović^d^, Jean-Ulrich Mullot^e^, Benjamin Misson^b^ and Jean-François Briand^a*^

^a^ *Université de Toulon, Laboratoire MAPIEM, EA 4323, Toulon, France*, ^b^ *Université de Toulon, Aix Marseille Université, CNRS, IRD, Mediterranean Institute of Oceanography (MIO), UM110, France, ^c^ Microbia environnement Observatoire Océanologique, Avenue Pierre Fabre 66650 Banyuls sur mer France,* ^d^ *Ruđer Bošković Institute, Division for Marine and Environmental Research, Bijenička 54, Zagreb, Croatia,* ^e^ *LASEM-Toulon, Base Navale De Toulon, BP 61, 83800 Toulon, France*

^*^: corresponding author; email : briand@univ-tln.fr

*^1^:*Present address: Centre for Microbial Ecology and Genomics, University of Pretoria, Pretoria, South Africa

1. **Supplementary materials**
   1. **16S rRNA gene amplicon sequencing of biofilm samples**

The PCR reaction (50 μL) contained 25 μL of 2X GoTaq® Long PCR Master Mix (Promega), 1 μM of each primer, and approximately 2 ng of DNA. The following thermal cycling scheme was used: initial denaturation at 95°C for 5 min, 30 cycles of denaturation at 95°C for 45 s, annealing at 50°C for 1min, followed by extension at 72°C for 1 min. The final extension was carried out at 72°C for 10 min. PCR products were checked on a 1.5% agarose gel. No amplification was yielded for negative controls consisting in the reaction mixture with MilliQ water as template.

- 1. **DNA extraction, 16S rRNA gene amplicon sequencing and quantification of seawater samples**

DNA was extracted from the Millipore filters by a combination of enzymatic cell lysis (Ghiglione et al., 2009) and AllPrep DNA/RNA Mini Kit (QIAGEN) according to the manufacturer’s instructions. The protocol for the DNA extraction is fully described in Coclet *et al.* (2019).

**REFERENCES**

Coclet, C., Garnier, C., Durrieu, G., Omanović, D., D’onofrio, S., Poupon, C. Le, et al. (2019). Changes in bacterioplankton communities resulting from direct and indirect interactions with trace metal gradients in an urbanized marine coastal area. *Front. Microbiol.* 10, 250. doi:10.3389/fmicb.2019.00257.

﻿Ghiglione JF, Conan P, Pujo-Pay M (2009) Diversity of total and active free-living vs. particle-attached bacteria in the euphotic zone of the NW Mediterranean Sea. FEMS Microbiol Lett 299:9–21. https://doi.org/10.1111/j.1574-6968.2009.01694.x

1. **Supplementary Figures**

**Fig. S1** Alpha-diversity descriptors including Simpson, Shannon, PD_Whole, Observed OTU, equitability and Chao1 calculated from 16S rRNA OTU table along each sampling sites (i.e. 6ext, MIS, Pt12, Pt15 and 41p) in surface (light) and bottom (dark) samples. Upper and lower lines correspond to the first and third quartile of the distribution of values. The median values are shown with horizontal black wide lines. Outliers are displayed as dots. *P-values* correspond to the results of one-way ANOVA analyses.

**Fig. S2** Biofilm densities (cell.cm^2^) in surface (light) and bottom (dark) polycarbonate plates along the different sampling sites determined by qPCR. *P-values* and “a,” “b,” “c” indexes correspond to the results of one-way ANOVA analyses and post hoc tests (HSD Tukey’s test), respectively.

**Fig. S3** Nonmetric dimensional scaling (nMDS) ordination based on Bray-Curtis dissimilarity for normalized 16S rRNA gene libraries by 16S copy number (A), for 16S rRNA gene libraries (B), and for level 3 of KEGG metabolic pathways (C) between the different sampling sites and sampling depths.

**Fig. S4** Bar graph representing relative proportions of significant families in bottom (blue) and surface (yellow) biofilm samples. Extended error bar plots showed pairwise comparison of significant family proportions (Welch’s t-test; *p* < 0.05) between bottom (blue) and surface (yellow) biofilm samples. Corrected p-value is determined using Fisher’s exact test.

**Fig. S5** Bar graph representing relative proportions of significant families between the sampling sites (41p: green; Pt15: blue; Pt12: yellow; MIS: grey; 6ext: red) in surface (A) and bottom biofilm samples. Extended error bar plots showed pairwise comparison of significant family proportions (Welch’s t-test; *p* < 0.01) between sites in surface (A) and bottom (B) biofilm samples. Corrected p-value is determined using Fisher’s exact test.

**Fig S6** Alpha-diversity descriptors including Simpson, Shannon, PD_Whole, Observed OTU, equitability and Chao1 calculated from 16S rRNA OTU table along each sampling sites (i.e. 6ext, MIS, Pt12, Pt15 and 41p) in seawater (blue) and biofilm (green) samples. Upper and lower lines correspond to the first and third quartile of the distribution of values. The median values are shown with horizontal black wide lines. Outliers are displayed as dots.

**Fig. S7** Bar graph representing relative proportions of significant families in seawater (blue) and biofilm (green) samples. Extended error bar plots showed pairwise comparison of significant family proportions (Welch’s t-test; *p* < 0.01) between seawater (blue) and biofilm (green) samples. Corrected p-value is determined using Fisher’s exact test.

**Fig. S8** Bar graph representing relative proportions of significant KEGG Pathways at level 2 of SEED metabolic hierarch in biofilms (green) and seawater (blue). Extended error bar plots showed pairwise comparison of significant KEGG Pathway proportions (Welch’s t-test; *p* < 0.01) at level 2 of SEED metabolic hierarchy between biofilms (green) and seawater (blue). Corrected p-value is determined using Fisher’s exact test.

**Fig. S9** Bar graph representing relative proportions of significant KEGG Pathways at level 2 of SEED metabolic hierarchy in biofilms from contaminated (6ext; red) and uncontaminated (41p; green) sites. Extended error bar plots showed pairwise comparison of significant KEGG Pathway proportions (Welch’s t-test; *p* < 0.01) at level 2 of SEED metabolic hierarchy between biofilms from contaminated (6ext; red) and uncontaminated (41p; green) sites. Corrected p-value is determined using Fisher’s exact test.

**Fig. S1** Alpha-diversity descriptors including Simpson, Shannon, PD_Whole, Observed OTU, equitability and Chao1 calculated from 16S rRNA OTU table along each sampling sites (i.e. 6ext, MIS, Pt12, Pt15 and 41p) in surface (light) and bottom (dark) samples. Upper and lower lines correspond to the first and third quartile of the distribution of values. The median values are shown with horizontal black wide lines. Outliers are displayed as dots. *P-values* correspond to the results of one-way ANOVA analyses.

**Fig. S2** Biofilm densities (cell.cm^2^) in surface (light) and bottom (dark) polycarbonate plates along the different sampling sites determined by qPCR. *P-values* and “a,” “b,” “c” indexes correspond to the results of one-way ANOVA analyses and post hoc tests (HSD Tukey’s test), respectively.

| A |  |
| --- | --- |
| B |  |
| C |  |

**Fig. S3** Nonmetric dimensional scaling (nMDS) ordination based on Bray-Curtis dissimilarity for normalized 16S rRNA gene libraries by 16S copy number (A), for 16S rRNA gene libraries (B), and for level 3 of KEGG metabolic pathways (C) between the different sampling sites and sampling depths.

**Fig. S4** Bar graph representing relative proportions of significant families in bottom (blue) and surface (yellow) biofilm samples. Extended error bar plots showed pairwise comparison of significant family proportions (Welch’s t-test; *p* < 0.05) between bottom (blue) and surface (yellow) biofilm samples. Corrected p-value is determined using Fisher’s exact test.

| A |  |
| --- | --- |
|  |  |
|  |  |
|  |  |
|  |  |
|  |  |
|  |  |

| B |  | |
| --- | --- | --- |
|  |  |  |
|  |  |  |
|  |  |  |
|  |  |  |
|  |  |  |
|  |  |  |
|  |  |  |
|  |  |  |
|  |  |  |
|  |  |  |
|  |  |  |
|  |  |  |
|  |  |  |
|  |  |  |

**Fig. S5** Bar graph representing relative proportions of significant families between the sampling sites (41p: green; Pt15: blue; Pt12: yellow; MIS: grey; 6ext: red) in surface (A) and bottom biofilm samples. Extended error bar plots showed pairwise comparison of significant family proportions (Welch’s t-test; *p* < 0.01) between sites in surface (A) and bottom (B) biofilm samples. Corrected p-value is determined using Fisher’s exact test.

**Fig S6** Alpha-diversity descriptors including Simpson, Shannon, PD_Whole, Observed OTU, equitability and Chao1 calculated from 16S rRNA OTU table along each sampling sites (i.e. 6ext, MIS, Pt12, Pt15 and 41p) in seawater (blue) and biofilm (green) samples. Upper and lower lines correspond to the first and third quartile of the distribution of values. The median values are shown with horizontal black wide lines. Outliers are displayed as dots.

**Fig. S7** Bar graph representing relative proportions of significant families in seawater (blue) and biofilm (green) samples. Extended error bar plots showed pairwise comparison of significant family proportions (Welch’s t-test; *p* < 0.01) between seawater (blue) and biofilm (green) samples. Corrected p-value is determined using Fisher’s exact test.

**Fig. S8** Bar graph representing relative proportions of significant KEGG Pathways at level 2 of SEED metabolic hierarch in biofilms (green) and seawater (blue). Extended error bar plots showed pairwise comparison of significant KEGG Pathway proportions (Welch’s t-test; *p* < 0.01) at level 2 of SEED metabolic hierarchy between biofilms (green) and seawater (blue). Corrected p-value is determined using Fisher’s exact test.

|  | **** |
| --- | --- |

**Fig. S9** Bar graph representing relative proportions of significant KEGG Pathways at level 2 of SEED metabolic hierarchy in biofilms from contaminated (6ext; red) and uncontaminated (41p; green) sites. Extended error bar plots showed pairwise comparison of significant KEGG Pathway proportions (Welch’s t-test; *p* < 0.01) at level 2 of SEED metabolic hierarchy between biofilms from contaminated (6ext; red) and uncontaminated (41p; green) sites. Corrected p-value is determined using Fisher’s exact test.

1. **Supplementary tables**

**Table S1.** Samples characteristics in Toulon Bay.

**Table S2.** Environmental (biotic and abiotic) characteristics of seawater samples in June 2015. The values represent the mean between sampling dates (n = 5) and the standard deviation for each parameter.

**Table S3.** Permutational multivariate analysis of variance (PERMANOVA) examining the effects of the factors “Site”, “Lifestyle” and “Depth” on the bacterial communities. Key to abbreviations and column headings: D.f, degrees of freedom; MS, mean square; F, F ratio; R2, coefficient of determination; P, p-value. Probabilities are marked as follows ‘***’ p<0.001; ‘**’ p<0.01; ‘*’ p<0.05.

**Table S4.** Permutational multivariate analysis of variance (PERMANOVA) examining the effects of the factors “Site”, and “Depth” on the normalized prokaryotic communities by cell densities. Key to abbreviations and column headings: D.f, degrees of freedom; MS, mean square; F, F ratio; R2, coefficient of determination; P, p-value. Probabilities are marked as follows ‘***’ p<0.001; ‘**’ p<0.01; ‘*’ p<0.05.

**Table S5.** Permutational multivariate analysis of variance (PERMANOVA) examining the effects of the factors “Site”, and “Depth” on functional profiles of KEGG Pathway at level 3 of SEED metabolic hierarchy. Key to abbreviations and column headings: D.f, degrees of freedom; MS, mean square; F, F ratio; R2, coefficient of determination; P, p-value. Probabilities are marked as follows ‘***’ p<0.001; ‘**’ p<0.01; ‘*’ p<0.05.

**Table S1.** Samples characteristics in Toulon Bay.

| **Sample name** | **Site** | **Depth** | **Latitude (°N)** | **Longitude (°E)** | **No. of raw amplicons** | **No. of amplicons after filtering** | **No. of OTUs** |
| --- | --- | --- | --- | --- | --- | --- | --- |
| 41p_S | 41p | Surface | 43° 4'21.74" | 5°57'21.48" | 31090 | 24391 | 470 |
| 41p_S1 | 41p | Surface | 43° 4'21.74" | 5°57'21.48" | 19130 | 15186 | 1273 |
| 41p_S2 | 41p | Surface | 43° 4'21.74" | 5°57'21.48" | 24135 | 19041 | 1349 |
| 41p_S3 | 41p | Surface | 43° 4'21.74" | 5°57'21.48" | 38830 | 28240 | 1442 |
| 41p_B | 41p | Bottom | 43° 4'21.74" | 5°57'21.48" | 27021 | 21754 | 439 |
| 41p_B1 | 41p | Bottom | 43° 4'21.74" | 5°57'21.48" | 41518 | 29254 | 1662 |
| 41p_B2 | 41p | Bottom | 43° 4'21.74" | 5°57'21.48" | 38056 | 15811 | 1537 |
| 41p_B3 | 41p | Bottom | 43° 4'21.74" | 5°57'21.48" | 29235 | 20540 | 1640 |
| Pt15_S | Pt15 | Surface | 43° 5'24.82" | 5°54'39.05" | 48244 | 36675 | 441 |
| Pt15_S1 | Pt15 | Surface | 43° 5'24.82" | 5°54'39.05" | 65410 | 37044 | 1739 |
| Pt15_S2 | Pt15 | Surface | 43° 5'24.82" | 5°54'39.05" | 42561 | 31438 | 1637 |
| Pt15_S3 | Pt15 | Surface | 43° 5'24.82" | 5°54'39.05" | 43641 | 30362 | 1428 |
| Pt15_B | Pt15 | Bottom | 43° 5'24.82" | 5°54'39.05" | 28125 | 21500 | 424 |
| Pt15_B1 | Pt15 | Bottom | 43° 5'24.82" | 5°54'39.05" | 20433 | 10336 | 1719 |
| Pt15_B2 | Pt15 | Bottom | 43° 5'24.82" | 5°54'39.05" | 25131 | 8647 | 1737 |
| Pt15_B3 | Pt15 | Bottom | 43° 5'24.82" | 5°54'39.05" | 27684 | 15758 | 1989 |
| Pt12_S | Pt12 | Surface | 43° 6'34.96" | 5°55'41.09" | 23279 | 17308 | 406 |
| Pt12_S1 | Pt12 | Surface | 43° 6'34.96" | 5°55'41.09" | 18631 | 12258 | 1450 |
| Pt12_S2 | Pt12 | Surface | 43° 6'34.96" | 5°55'41.09" | 15387 | 10164 | 1438 |
| Pt12_S3 | Pt12 | Surface | 43° 6'34.96" | 5°55'41.09" | 22721 | 16207 | 1244 |
| Pt12_B | Pt12 | Bottom | 43° 6'34.96" | 5°55'41.09" | 38367 | 26656 | 469 |
| Pt12_B1 | Pt12 | Bottom | 43° 6'34.96" | 5°55'41.09" | 25947 | 19365 | 1666 |
| Pt12_B2 | Pt12 | Bottom | 43° 6'34.96" | 5°55'41.09" | 25992 | 18822 | 1578 |
| Pt12_B3 | Pt12 | Bottom | 43° 6'34.96" | 5°55'41.09" | 30137 | 18778 | 1738 |
| MIS_S | MIS | Surface | 43° 7'9.37" | 5°54'40.07" | 26720 | 21205 | 442 |
| MIS_S1 | MIS | Surface | 43° 7'9.37" | 5°54'40.07" | 11297 | 5828 | 782 |
| MIS_S2 | MIS | Surface | 43° 7'9.37" | 5°54'40.07" | 19638 | 11971 | 1100 |
| MIS_S3 | MIS | Surface | 43° 7'9.37" | 5°54'40.07" | 10571 | 6279 | 760 |
| MIS_B | MIS | Bottom | 43° 7'9.37" | 5°54'40.07" | 32551 | 25934 | 412 |
| MIS_B1 | MIS | Bottom | 43° 7'9.37" | 5°54'40.07" | 34347 | 25032 | 1663 |
| MIS_B2 | MIS | Bottom | 43° 7'9.37" | 5°54'40.07" | 19239 | 14098 | 1676 |
| MIS_B3 | MIS | Bottom | 43° 7'9.37" | 5°54'40.07" | 16679 | 11618 | 1780 |
| 6ext_S | 6ext | Surface | 43° 7'8.65" | 5°54'57.31" | 31290 | 22615 | 415 |
| 6ext_S1 | 6ext | Surface | 43° 7'8.65" | 5°54'57.31" | 22281 | 15624 | 1608 |
| 6ext_S2 | 6ext | Surface | 43° 7'8.65" | 5°54'57.31" | 32959 | 22598 | 1677 |
| 6ext_S3 | 6ext | Surface | 43° 7'8.65" | 5°54'57.31" | 43457 | 24926 | 1695 |
| 6ext_B | 6ext | Bottom | 43° 7'8.65" | 5°54'57.31" | 28337 | 21647 | 496 |
| 6ext_B1 | 6ext | Bottom | 43° 7'8.65" | 5°54'57.31" | 24539 | 17898 | 1639 |
| 6ext_B2 | 6ext | Bottom | 43° 7'8.65" | 5°54'57.31" | 25837 | 17138 | 1690 |
| 6ext_B3 | 6ext | Bottom | 43° 7'8.65" | 5°54'57.31" | 33373 | 17531 | 1727 |

**Table S2.** Environmental (biotic and abiotic) characteristics of seawater samples in June 2015. The values represent the mean between sampling dates (n = 5) and the standard deviation for each parameter.

| Site | **41p** | | **Pt15** | | **Pt12** | | **MIS** | | **6ext** | |
| --- | --- | --- | --- | --- | --- | --- | --- | --- | --- | --- |
| Depth | Surface | Bottom | Surface | Bottom | Surface | Bottom | Surface | Bottom | Surface | Bottom |
| **Temperature** | 22 ± 1.4 | 19 ± 1.7 | 22 ± 1.6 | 21 ± 1.5 | 22 ± 1.6 | 21 ± 1.4 | 23 ± 1.3 | 21 ± 1.4 | 23 ± 1.3 | 21 ± 1.8 |
| **Salinity** | 39 ± 0.13 | 39 ± 0.05 | 39 ± 0.49 | 39 ± 0.15 | 39 ± 0.58 | 39 ± 0.15 | 38 ± 0.68 | 39 ± 0.07 | 38 ± 0.60 | 39 ± 0.09 |
| **Chlorophyll *a*** | 0.20 ± 0.06 | 0.37 ± 0.11 | 0.89 ± 0.30 | 1.3 ± 0.20 | 0.90 ± 0.57 | 2.0 ± 1.0 | 0.99 ± 0.31 | 2.6 ± 1.1 | 1.2 ± 0.51 | 3.5 ± 1.8 |
| **DOC** | 1.2 ± 0.14 | 1.1 ± 0.09 | 1.2 ± 0.15 | 1.2 ± 0.10 | 1.3 ± 0.12 | 1.2 ± 0.10 | 1.3 ± 0.24 | 1.2 ± 0.10 | 1.4 ± 0.16 | 1.2 ± 0.08 |
| **TN** | 0.08 ± 0.04 | 0.06 ± 0.02 | 0.08 ± 0.01 | 0.07 ± 0.02 | 0.09 ± 0.04 | 0.07 ± 0.02 | 0.15 ± 0.04 | 0.08 ± 0.02 | 0.11 ± 0.04 | 0.09 ± 0.02 |
| **Al** | 4788 ± 4436 | 4852 ± 4581 | 4419 ± 4955 | 4483 ± 5136 | 4742 ± 5191 | 4312 ± 5093 | 4157 ± 4651 | 4142 ± 4630 | 4216 ± 4475 | 3472 ± 4969 |
| **As** | 25 ± 2.4 | 27 ± 4.4 | 28 ± 2.7 | 27 ± 4.4 | 25 ± 5.0 | 24 ± 4.7 | 25 ± 4.9 | 26 ± 3.0 | 30 ± 3.8 | 27 ± 5.9 |
| **Ba** | 59 ± 2.2 | 59 ± 2.9 | 61 ± 2.3 | 60 ± 2.1 | 61 ± 2.5 | 62 ± 7.9 | 63 ± 3.7 | 60 ± 2.1 | 69 ± 4.0 | 68 ± 8.3 |
| **Be** | 46 ± 25 | 45 ± 26 | 48 ± 26 | 44 ± 5.9 | 49 ± 19 | 48 ± 14 | 52 ± 12 | 59 ± 28 | 63 ± 32 | 63 ± 33 |
| **Cd** | 0.063 ± 0.017 | 0.060 ± 0.022 | 0.088 ± 0.017 | 0.083 ± 0.0081 | 0.12 ± 0.029 | 0.13 ± 0.11 | 0.14 ± 0.056 | 0.13 ± 0.053 | 0.15 ± 0.019 | 0.17 ± 0.043 |
| **Cr** | 7.2 ± 4.7 | 7.0 ± 2.7 | 7.1 ± 3.4 | 7.0 ± 1.9 | 6.7 ± 2.1 | 8.0 ± 3.5 | 7.7 ± 2.0 | 6.4 ± 2.4 | 6.1 ± 1.4 | 5.0 ± 1.1 |
| **Cs** | 2.5 ± 0.10 | 2.5 ± 0.12 | 2.5 ± 0.09 | 2.5 ± 0.09 | 2.5 ± 0.09 | 2.5 ± 0.07 | 2.5 ± 0.13 | 2.5 ± 0.04 | 2.6 ± 0.11 | 2.5 ± 0.05 |
| **Cu** | 5.1 ± 1.2 | 4.2 ± 0.95 | 25 ± 6.6 | 12 ± 1.8 | 34 ± 7.9 | 30 ± 23 | 72± 19 | 28 ± 16 | 93 ± 22 | 26 ± 10 |
| **Fe** | 64 ± 41 | 64 ± 48 | 68 ± 50 | 130 ± 92 | 63 ± 52 | 66 ± 54 | 63 ± 42 | 66 ± 39 | 70 ± 37 | 69 ± 46 |
| **Li** | 28806 ± 2049 | 28921 ± 3253 | 29658 ± 4865 | 29403 ± 4373 | 29393 ± 4157 | 28757 ± 4732 | 27747 ± 3817 | 28355 ± 3057 | 28235 ± 2619 | 27813 ± 2526 |
| **Mn** | 14 ± 1.8 | 14 ± 4.3 | 24 ± 7.9 | 24 ± 7.1 | 25 ± 7.2 | 28 ± 14 | 26 ± 7.5 | 24 ± 6.0 | 30 ± 5.6 | 48 ± 6.5 |
| **Mo95** | 164 ± 9.6 | 162 ± 7.1 | 162 ± 8.7 | 161 ± 7.4 | 160 ± 7.5 | 160 ± 4.9 | 159 ± 6.0 | 162 ± 6.1 | 164 ± 8.7 | 161 ± 5.0 |

**Table S2.** Continued

| Site | **41p** | | **Pt15** | | **Pt12** | | | **MIS** | | **6ext** | |
| --- | --- | --- | --- | --- | --- | --- | --- | --- | --- | --- | --- |
| Depth | Surface | Bottom | Surface | Bottom | | Surface | Bottom | Surface | Bottom | Surface | Bottom |
| **Pb** | 0.26 ± 0.13 | 0.32 ± 0.086 | 1.1 ± 0.19 | 0.65 ± 0.17 | | 1.5 ± 0.35 | 2.2 ± 2.3 | 1.4 ± 0.19 | 1.7 ± 0.18 | 4.6 ± 0.87 | 7.2 ± 6.1 |
| **Rb** | 1476 ± 20 | 1477 ± 46 | 1479 ± 73 | 1475 ± 61 | | 1468.60 ± 56.33 | 1461 ± 63 | 1437 ± 54 | 1460 ± 40 | 1460 ± 49 | 1455 ± 35 |
| **Sb** | 3.6 ± 0.27 | 3.5 ± 0.23 | 4.1 ± 0.79 | 4.0 ± 0.79 | | 4.1 ± 0.69 | 4.6 ± 1.3 | 4.3 ± 0.90 | 4.2 ± 0.84 | 5.4 ± 0.82 | 5.9 ± 1.9 |
| **Sn** | 1.7 ± 0.40 | 1.8 ± 0.46 | 1.8 ± 0.49 | 1.7 ± 0.48 | | 2.2 ± 1.6 | 1.7 ± 0.56 | 1.7 ± 0.50 | 1.6 ± 0.45 | 1.9 ± 0.48 | 1.8 ± 0.37 |
| **Sr** | 106041 ± 1881 | 105845 ± 3392 | 106071 ± 4756 | 105787 ± 3868 | | 105608 ± 4101 | 104734 ± 3787 | 103183 ± 4011 | 104936 ± 2494 | 104297 ± 3280 | 104835 ± 2195 |
| **Ti** | 16 ± 12 | 13 ± 10 | 16 ± 13 | 13 ± 12 | | 14 ± 14 | 13 ± 15 | 15 ± 15 | 17 ± 15 | 14 ± 12 | 13 ± 15 |
| **U** | 16 ± 1.3 | 15 ± 1.6 | 15 ± 2.0 | 15 ± 1.9 | | 15 ± 1.7 | 15 ± 2.1 | 15 ± 1.4 | 16 ± 1.3 | 16 ± 1.1 | 16 ± 0.40 |
| **V** | 43 ± 2.2 | 44 ± 4.5 | 44 ± 5.2 | 44 ± 4.1 | | 43 ± 4.6 | 42 ± 4.4 | 42 ± 4.3 | 42 ± 3.4 | 43 ± 4.1 | 42 ± 3.4 |
| **Zn** | 18 ± 8.5 | 15 ± 5.1 | 49 ± 10 | 24 ± 4.7 | | 61 ± 13 | 108 ± 152 | 130 ± 28 | 70 ± 38 | 290 ± 46 | 166 ± 81 |

**Table S3.** Permutational multivariate analysis of variance (PERMANOVA) examining the effects of the factors “Site”, “Lifestyle” and “Depth” on the prokaryotic communities. Key to abbreviations and column headings: D.f, degrees of freedom; MS, mean square; F, F ratio; R2, coefficient of determination; P, p-value. Probabilities are marked as follows ‘***’ p<0.001; ‘**’ p<0.01; ‘*’ p<0.05.

| Taxonomic rank | Source of variation | D.f | MS | F | R2 | P | Significance |
| --- | --- | --- | --- | --- | --- | --- | --- |
| Phylum | Site | 4 | 0.019737 | 7.490 | 0.13659 | 0.001 | *** |
|  | Lifestyle | 1 | 0.224852 | 85.326 | 0.38903 | 0.001 | *** |
|  | Depth | 1 | 0.029882 | 11.339 | 0.05170 | 0.002 | ** |
|  | Site:Lifestyle | 4 | 0.027082 | 10.277 | 0.18743 | 0.001 | *** |
|  | Site:Depth | 4 | 0.012060 | 4.577 | 0.08346 | 0.001 | *** |
|  | Lifestyle:Depth | 1 | 0.010746 | 4.078 | 0.01859 | 0.020 | * |
|  | Site:Lifestyle:Depth | 4 | 0.006069 | 2.303 | 0.04200 | 0.015 | * |
|  | Residuals | 20 | 0.002635 | 0.09119 |  |  |  |
| Class | Site | 4 | 0.04588 | 8.316 | 0.13310 | 0.001 | *** |
|  | Lifestyle | 1 | 0.55500 | 100.607 | 0.40255 | 0.001 | *** |
|  | Depth | 1 | 0.10884 | 19.730 | 0.07894 | 0.001 | *** |
|  | Site:Lifestyle | 4 | 0.04704 | 8.528 | 0.13648 | 0.001 | *** |
|  | Site:Depth | 4 | 0.03424 | 6.206 | 0.09933 | 0.001 | *** |
|  | Lifestyle:Depth | 1 | 0.05365 | 9.726 | 0.03891 | 0.001 | *** |
|  | Site:Lifestyle:Depth | 4 | 0.01057 | 1.916 | 0.03066 | 0.045 | * |
|  | Residuals | 20 | 0.00552 | 0.08002 |  |  |  |
| Order | Site | 4 | 0.05791 | 9.147 | 0.09456 | 0.001 | *** |
|  | Lifestyle | 1 | 1.41571 | 223.626 | 0.57794 | 0.001 | *** |
|  | Depth | 1 | 0.11711 | 18.499 | 0.04781 | 0.001 | *** |
|  | Site:Lifestyle | 4 | 0.07389 | 11.672 | 0.12066 | 0.001 | *** |
|  | Site:Depth | 4 | 0.03965 | 6.264 | 0.06475 | 0.001 | *** |
|  | Lifestyle:Depth | 1 | 0.04722 | 7.460 | 0.01928 | 0.002 | ** |
|  | Site:Lifestyle:Depth | 4 | 0.01428 | 2.255 | 0.02332 | 0.039 | * |
|  | Residuals | 20 | 0.00633 | 0.05169 |  |  |  |
| Family | Site | 4 | 0.04076 | 6.820 | 0.08150 | 0.001 | *** |
|  | Lifestyle | 1 | 1.12012 | 187.399 | 0.55986 | 0.001 | *** |
|  | Depth | 1 | 0.11508 | 19.253 | 0.05752 | 0.001 | *** |
|  | Site:Lifestyle | 4 | 0.06283 | 10.512 | 0.12562 | 0.001 | *** |
|  | Site:Depth | 4 | 0.03443 | 5.760 | 0.06883 | 0.001 | *** |
|  | Lifestyle:Depth | 1 | 0.04781 | 7.998 | 0.02390 | 0.002 | ** |
|  | Site:Lifestyle:Depth | 4 | 0.01152 | 1.927 | 0.02303 | 0.054 | . |
|  | Residuals | 20 | 0.00598 | 0.05975 |  |  |  |

**Table S3.** Continued

| Taxonomic rank | Source of variation | D.f | MS | F | R2 | P | Significance |
| --- | --- | --- | --- | --- | --- | --- | --- |
| Genus | Site | 4 | 0.04057 | 9.369 | 0.08104 | 0.001 | *** |
|  | Lifestyle | 1 | 1.31058 | 302.628 | 0.65437 | 0.001 | *** |
|  | Depth | 1 | 0.13668 | 31.562 | 0.06825 | 0.001 | *** |
|  | Site:Lifestyle | 4 | 0.02516 | 5.809 | 0.05024 | 0.001 | *** |
|  | Site:Depth | 4 | 0.02733 | 6.310 | 0.05458 | 0.001 | *** |
|  | Lifestyle:Depth | 1 | 0.06001 | 13.858 | 0.02996 | 0.001 | *** |
|  | Site:Lifestyle:Depth | 4 | 0.00917 | 2.117 | 0.01831 | 0.037 | * |
|  | Residuals | 20 | 0.00433 | 0.04325 |  |  |  |

**Table S4.** Permutational multivariate analysis of variance (PERMANOVA) examining the effects of the factors “Site”, and “Depth” on the normalized prokaryotic communities by cell densities. Key to abbreviations and column headings: D.f, degrees of freedom; MS, mean square; F, F ratio; R2, coefficient of determination; P, p-value. Probabilities are marked as follows ‘***’ p<0.001; ‘**’ p<0.01; ‘*’ p<0.05.

|  | Source of variation | D.f | MS | F | R2 | P | Significance |
| --- | --- | --- | --- | --- | --- | --- | --- |
| Level 3 | Site | 4 | 0.392 | 3.5430 | 0.293 | 0.001 | *** |
|  | Depth | 1 | 0.184 | 1.6652 | 0.034 | 0.188 | NS |
|  | Site:Depth | 4 | 0.349 | 3.1529 | 0.260 | 0.001 | *** |
|  | Residuals | 20 | 0.111 |  |  |  |  |

**Table S5.** Permutational multivariate analysis of variance (PERMANOVA) examining the effects of the factors “Site”, and “Depth” on functional profiles of KEGG Pathway at level 3 of SEED metabolic hierarchy. Key to abbreviations and column headings: D.f, degrees of freedom; MS, mean square; F, F ratio; R2, coefficient of determination; P, p-value. Probabilities are marked as follows ‘***’ p<0.001; ‘**’ p<0.01; ‘*’ p<0.05.

|  | Source of variation | D.f | MS | F | R2 | P | Significance |
| --- | --- | --- | --- | --- | --- | --- | --- |
| Level 3 | Site | 4 | 0.0006 | 5.21 | 0.34 | 0.001 | *** |
|  | Depth | 1 | 0.0009 | 8.24 | 0.13 | 0.002 | ** |
|  | Site:Depth | 4 | 0.0003 | 3.07 | 0.20 | 0.003 | ** |
|  | Residuals | 20 | 0.0001 |  |  |  |  |
